# Protein-restricted diet during pregnancy after insemination alters behavioral phenotypes of the progeny

**DOI:** 10.1186/s12263-016-0550-2

**Published:** 2017-01-19

**Authors:** Tamio Furuse, Kunio Miyake, Takashi Kohda, Hideki Kaneda, Takae Hirasawa, Ikuko Yamada, Tomoko Kushida, Misho Kashimura, Kimio Kobayashi, Fumitoshi Ishino, Takeo Kubota, Shigeharu Wakana

**Affiliations:** 1 Japan mouse clinic, RIKEN BRC, 3-1-1 Koyadai, Tsukuba, Ibaraki 305-0074 Japan; 2 Department of Epigenetic Medicine, University of Yamanashi, 1110 Shimokato, Chuo, Yamanashi 409-3898 Japan; 3 Department of Epigenetics, Medical Research Institute, Tokyo Medical and Dental University, 1-5-45 Yushima, Bunkyoku, Tokyo, 113-8510 Japan

**Keywords:** Maternal nutrition, In vitro fertilization, Behavioral phenotypes, Gene expression and DNA methylation

## Abstract

**Background:**

Epidemiological studies suggest that hyponutrition during the fetal period increases the risk of mental disorders such as attention deficit hyperactivity disorder and autism-spectrum disorder, which has been experimentally supported using animal models. However, previous experimental hyponutrition or protein-restricted (PR) diets affected stages other than the fetal stage, such as formation of the egg before insemination, milk composition during lactation, and maternal nursing behavior.

**Results:**

We conducted in vitro fertilization and embryo transfer in mice and allowed PR diet and folic acid-supplemented PR diet to affect only fetal environments. Comprehensive phenotyping of PR and control-diet progenies showed moderate differences in fear/anxiety-like, novelty-seeking, and prosocial behaviors, irrespective of folic-acid supplementation. Changes were also detected in gene expression and genomic methylation in the brain.

**Conclusions:**

These results suggest that epigenetic factors in the embryo/fetus influence behavioral and epigenetic phenotypes of progenies. Significant epigenetic alterations in the brains of the progenies induced by the maternal-protein restriction were observed in the present study. To our knowledge, this is first study to evaluate the effect of maternal hyponutrition on behavioral phenotypes using reproductive technology.

**Electronic supplementary material:**

The online version of this article (doi:10.1186/s12263-016-0550-2) contains supplementary material, which is available to authorized users.

## Background

Genetic factors are known to influence susceptibility to psychiatric disorders, including schizophrenia, bipolar disorder, major depressive disorder, autism-spectrum disorder (ASD), and attention deficit hyperactivity disorder (AD/HD) [1]. However, recent studies have revealed that environmental and nutritional factors also affect neuronal functions, probably via epigenetic mechanisms [2–5]. In the field of metabolic diseases, “Developmental Origins of Health and Disease” (DOHaD) is the concept that in utero experiences reprogram susceptibility to adult metabolic diseases [6]. It is of interest to test if this concept is applicable to behavioral symptoms in developmental disorders such as AD/HD and ASD. Several studies using rodent models have been conducted to evaluate the effects of maternal hyponutrition on phenotypes of progenies. Restricted feeding in mothers of mice or rats caused body weight restriction, developmental anomalies, changes in metabolic phenotypes, patterns of gene expression, and DNA methylation in progenies [7]. Isocaloric diets, such as the protein restriction (PR) model, are fed in an alternative restriction of the diet [8, 9] by compensating shortage of energy source with carbohydrates [8]. Hence, protein-carbohydrate balance is altered in the PR, making it suitable for evaluating the effects of maternal nutritional imbalance, and not that of calorie shortage [10]. Several reports have examined the interactions between maternal-protein restriction and adult phenotypic/epigenetic alterations [8, 11–13].

Maternal PR diet, however, affects not only fetal environments but also formation of the egg before insemination, nutritional condition of maternal milk, and maternal nursing behavior, and therefore, it has potential confounding variables at different stages. In the present study, we performed in vitro fertilization (IVF) to produce neonates and then obtained progenies that were adopted by foster mothers before weaning. This allowed the PR diet to affect only the fetal stage of development.

## Methods

### Ethics statement of animal experiments

All procedures described here were reviewed and approved by the Institutional Animal Care and Use Committee of RIKEN Tsukuba Branch and were performed in accordance with the RIKEN Guiding Principles for the Care and Use of Laboratory Animals (No. 10-013).

### Experimental design

**Fig. 1:**
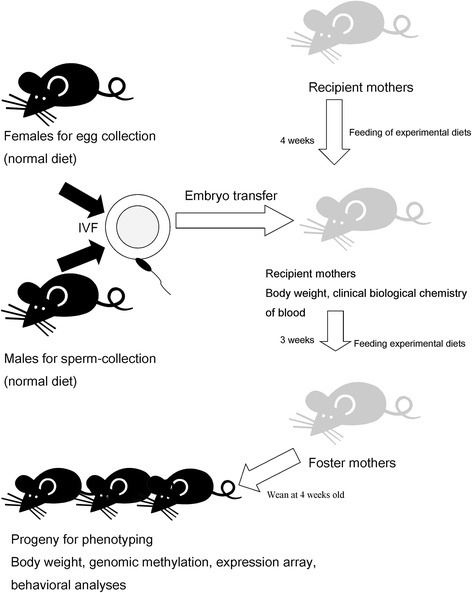
Experimental strategy. The experimental design of this study is briefly described. Normal diet (CE-2) was fed to females used for oocyte collection and males used for sperm collection. Recipient mothers consumed the control diet (CD) or experimental diet (PR and FA) for 1 month before embryo transfer. Embryos were generated by fertilizing the eggs with sperm of C57BL/6J (B6) mice. After cesarean-section delivery, neonates were fostered by ICR females that had consumed CD, PR, or FA diets. After weaning, offspring consumed the control diet (CD). Serum was separated from blood taken from the ICR females. Brain tissue was collected from offspring after behavioral tests

The experimental design is described briefly in Fig. 1. IVF and embryo transfer (ET) techniques were used for mouse production. Female ICR mice, an outbred stock from the Swiss mouse developed in the Institute of Cancer Research [14], were used as recipient and foster mothers. Embryos were generated by fertilizing the eggs and sperm of C57BL/6J (B6) mice that had consumed a normal diet (CE-2, CLEA Japan, Inc., Tokyo, Japan). CE-2 is a rodent diet used for rearing and breeding. The diet consists of crude sources of protein (soybean waste, whitefish meal, and yeast), fat (cereal germ and soybean oil), and carbohydrate (wheat flour and corn). Detailed ingredients of the CE-2 diet are described on the webpage of the supplier (http://www.clea-japan.com/en/diets/diet_a/a_03.html). AIN93G control diet (CD), protein-restricted diet (PR), and protein-restricted diet supplemented with folic acid (FA) were used as experimental diets in recipient mothers beginning 1 month before ET until delivery. The nutrient sources of these experimental diets are described in Table 1. Neonates were adopted by ICR foster mothers immediately after cesarean section. After weaning, we provided the CD to the progenies. The body weight of the mothers and progenies, clinical biochemistry of the recipient mothers, gene expression in the brain of the progenies, and behavioral phenotypes of the progenies were examined.

**Table 1:** Components of experimental diets

	Composition (% weight)		
Component	CD	PR	FA
Vitamin-free casein	20	5.0	5.0
L-cystine	0.3	0.077	0.077
Corn starch	39.7486	51.1716	51.1716
Alpha-corn starch	13.2	17.0	17.0
Sucrose	10.0	10.0	10.0
Soy bean oil	7.0	7.0	7.0
Cellulose powder	5.0	5.0	5.0
AIN-93G mineral mixture	3.5	3.5	3.5
AIN-93 vitamin mixture	1.0	1.0	1.0
Folic acid	0.02	0.02	0.1
Choline bitartrate	0.25	0.25	0.25
Tertiary butylhydroquinone	0.0014	0.0014	0.0014
Total	100.0	100.0	100.0

*CD* control diet, *PR* protein-restricted diet, *FA* PR diet with supplemental-folic acid

### Experimental diets

The CD was very similar to the standard formation of AIN93G [15]. Test diets were modified from AIN93G formulation as follows: PR, ratio of vitamin-free casein was decreased from 20 to 5%; FA, 0.8 g/kg of folic acid was added to the PR (Table 1). The CD and PR contained 0.02% folic acid (weight composition) and the FA contained 0.1% folic acid (weight composition). Experimental diets were purchased from a commercial supplier (Oriental yeast Co. Ltd, Tokyo, Japan). These diets were isocaloric (4 kcal/g in each feed) and fed to the recipient mothers ad libitum. In addition, only the CD diet was fed to the foster mothers ad libitum.

### Mice

All mice used as recipient and foster mothers, as well as for egg and sperm collection, were purchased from a commercial supplier (CLEA Japan, Inc.).

### In vitro fertilization and embryo transfer

We conducted IVF and ET as previously described [16] to produce mice for behavioral tests, expression array, and DNA-methylation analysis of brains. In brief, sperm was collected from the caudae epididymides of 25-week-old B6 males and allowed to diffuse in fertilization medium. After preincubation for approximately 1 h to allow for capacitation, the sperm was used for insemination. Meanwhile, 3-week-old female mice (*N* = 7) were superovulated using intraperitoneal injections of PMSG and HCG (serotropin and gonatropin; ASKA Pharmaceutical Co., Tokyo, Japan) with an interval of 48 h between injections. Approximately 15–17 h after the HCG injection, the oocytes-cumulus complexes were collected from the oviducts of superovulated female B6 mice. Then, the complexes from several female mice were placed in fertilization medium. Insemination was performed by adding the preincubated sperm suspension to the fertilization medium containing complexes and cultured at 37 °C with 5% CO_2_. 24 h after insemination, 24 2-cell embryos were transferred into the oviducts of pseudopregnant ICR females (CLEA Japan, Inc.) mated to vasectomized ICR males. ICR females that consumed experimental diets were used as pseudopregnant recipients for the ET. Female ICR mice were purchased at 4 to 5 weeks of age, and introduced to the facility for experiments. These mice were split randomly into three groups; CD, PR, and FA. The foster mothers consumed the experimental diets beginning 4 weeks before ET and continuing until delivery. Body weight of recipient mice was measured once per week until delivery via cesarean section [17]. We used only male progeny for behavioral, genomic methylation, and gene expression analyses. Maternal separation in early life alters behavioral phenotypes, gene expression, and brain functions of mice [18, 19]. Hence, newborn male mice had to be adopted by foster mothers immediately after cesarean section (6–7 neonates/foster mother), and body weights were not measured. However, we measured body weights of female neonates to determine the effects of maternal hyponutrition on birth weight of offspring immediately after cesarean section. In order to reduce the number of foster mothers, only males were adopted by foster mothers.

### Clinical-biochemical test

We carried out a clinical-biochemical test using serum collected from ICR females at 13 weeks of age. The mice consumed experimental diets for 7 weeks. Blood collection, separation of serum, and analysis was performed as previously described [20]. In brief, we collected 200 μl of blood from the retro-orbital sinus using a Pasteur pipette (Thermo Fisher Scientific Inc., Waltham, MA). The collected blood was transferred to a microtube containing Coagulant Wako solution (Wako Pure Chemical Industries Co, Osaka, Japan) and serum was separated by centrifuging twice (600–2500×*g* for 5–15 min). We measured total protein (TP), urea nitrogen (UN), albumin (ALB), total cholesterol (T-CHO), HDL-cholesterol (HDL-C), triglyceride (TG), and LDL-cholesterol (LD-C) in the serum with an automatic clinical-biochemistry analyzer (JCA-BM2250, JEOL, Ltd., Tokyo, Japan). Number of subjects: CD, 7; PR, 7; FA, 6.

### DNA/RNA extraction

We harvested whole brains from adult male progenies (19 weeks of age) that were used in behavioral analyses, methylation analyses, and expression arrays. Adult males were euthanized via cervical dislocation. Brain samples were rinsed with PBS and frozen immediately using liquid nitrogen. The frozen tissues were stored at −80 °C. RNA and DNA were extracted simultaneously using an AllPrep DNA/RNA Mini Kit (Qiagen, Valencia, CA, USA) from the whole brains, including cerebrum and cerebellum. We then performed gene methylation and expression arrays as described below.

### Genomic methylation

Genome-wide DNA methylation analysis was performed as previously reported with slight modification [21]. Genomic DNA was fragmented with methylation-insensitive restriction enzymes (*Mse*I, *Bfa*I, and *CSP*6I) to yield fragments ranging in size from 200 to 1000 base pairs. The enrichment of methylated DNA was performed using the MethylMiner Methylated DNA Enrichment kit (Thermo Fisher Scientific Inc.). Briefly, CpG-dense methylated DNA was isolated from fragmented genomic DNA via binding to the methyl-CpG domain of the MBD2 protein, which is coupled to paramagnetic Dynabeads M-280 streptavidin via a biotin linker. A portion of the fragmented DNA (input) was left untreated and used as a control. DNA was amplified and cleaned using the GenomePlex Complete Whole Genome Amplification (WGA) Kit and GenElute PCR Clean-Up Kit (Sigma-Aldrich St. Louis, MO, USA). For analysis of genomic methylation, we used the Mouse Promoter 1.0R Array (Affymetrix) against methylated (IP) and input DNA (IN). Number of subjects: CD, 3; PR, 4; FA, 4.

### Expression array

Expression analysis by DNA microarray was performed with the Agilent system (G4846A; Whole Mouse Genome Microarray Kit (4x44K) Agilent Technologies, Santa Clara, CA, USA). The probes for the microarray were prepared and labeled by Cy3 according to the manufacturer’s protocol (Agilent Technologies). Arrays were scanned with a G2505C Microarray Scanner System (Agilent Technologies). Data were normalized using the *R* stats package with the qspline function of the “affy” package in the Bioconductor [22].

### Validity of the methylation and expression array data

Results of methylation array analyses have been validated in previous studies [21, 23]. In these studies, the array-based methylation quantification data showed strong concordance with results obtained from bisulfite sequencing (*r* = 0.81 to 0.88) [21, 24]. In addition, expression array data was also validated in previous studies. Expression data obtained by the one color microarray platform showed strong concordance with a TaqMan assay (*r* = 0.876) in a large scale microarray quality control project [25].

### Data availability

Expression and methylation array data were deposited in the NCBI gene expression omnibus (http://www.ncbi.nlm.nih.gov/geo/) and accession numbers of the datasets are as follows: GSE79847.

### Behavioral tests

We performed the following behavioral tests: open-field test (6-weeks-old), object-exploration test (7-weeks-old), social-interaction test (8-weeks-old), home-cage activity test (9 to 10-weeks-old), light/dark-transition test (11-weeks-old), fear-conditioning test (12-weeks-old), and tail-suspension test (13-weeks-old). Results of the behavioral tests are sensitive to prior experiences [26]. To suppress effects of prior experiences, the behavioral tests were performed in order of less stimulation as described above. We used male mice for the behavioral tests in order to eliminate the effects of the estrous cycle in females. Number of subjects: CD, 10; PR, 10; FA, 7.

#### Open-field test

Open-field test was performed as previously described [27]. Each mouse was placed in the corner of an open-field apparatus (400 mm wide × 400 mm long × 300 mm high; O’Hara & Co., Ltd., Tokyo, Japan) made of white polyvinyl chloride. The distance traveled by each animal in the open-field was recorded for 20 min with a video-imaging system (Image OF9; O’Hara & Co., Ltd.).

#### Object-exploration test

We performed an object-exploration test as previously described [27]. Briefly, each mouse was placed in the open-field apparatus without the novel object for 20 min of acclimation before testing. A transparent acrylic tube (bottom diameter, 66 mm; top diameter, 44 mm; height, 154 mm) containing marbles was placed in the center of the open-field. The total time spent exploring the object and frequency of exploration during a 10 min period was determined with a commercial video-imaging system (O’Hara & Co., Ltd.). Exploration was defined as a distance of < 1 cm between the object and the mouse.

#### Social-interaction test

A social-interaction test was performed as previously described [27]. We used a procedure similar to the object-exploration test to examine social-interaction behavior in mice. A mouse was positioned in the center of the open-field apparatus with an empty transparent acrylic tube and habituated for 20 min. Then, the empty tube was exchanged for another tube containing a novel male mouse, which was placed in the center of the open field. The total time spent exploring the novel mouse and the frequency of exploration during a 10-min period was determined by video-imaging.

#### Home-cage activity

A home-cage activity test was performed as previously described [27]. Each mouse was placed alone in a testing cage (227 × 329 × 133 mm) under a 12 h light–dark cycle (light on at 08:00 am) and had free access to both food and water. After 1 day of acclimation, spontaneous activity in the cage was measured for 5 days (starting at 08:00 am) using an infrared sensor (activity sensor, O’Hara & Co., Ltd.).

#### Light/dark transition test

A light/dark transition test was performed as described previously [27]. A commercially available light/dark chamber (O’Hara & Co., Ltd.) consisting of a light chamber (200 mm long × 200 mm wide × 250 mm high) made of white vinyl chloride plates and a dark chamber with the same dimensions made of black vinyl chloride plates was used. The apparatus had an opening (50 mm wide × 30 mm high) in the middle of the wall joining the two chambers. The opening was controlled by a guillotine door. The number of transitions between chambers, time spent in the light chamber, and the total distance traveled by the mouse were measured.

#### Fear-conditioning test

We performed a fear-conditioning test as previously described [28] with slight modification. On the training day (day 1), each mouse was placed in a shock chamber with white walls (O’Hara & Co. Ltd.) (box A) and 120 s later, 4 tone-shock pairs were given at 90 s intervals. Each tone-shock pair consisted of a tone (70 dB, 10 kHz) for 30 s and a foot shock for 2 s at 0.5 mA. The foot shock was presented to the mice during the last 2 s of the tone. On day 2, each mouse was placed back in box A for 6 min to measure contextual freezing. On day 3, each mouse was placed in a white transparent chamber (box B), and 180 s later, 180 s tones were delivered. Freezing was measured during the first 180 s in box B, representing the response to an unconditioned context, and then during the next 180 s in the presence of the conditioned tone.

#### Tail-suspension test

In order to examine depression-like behavior in the progenies, a tail-suspension test was performed as described previously [29] with slight modifications. The tail of the mouse was fixed to a metal plate using adhesive tape. Then, the mouse was suspended from the metal plate in an experimental box (400 mm × 400 mm × 300 mm; O’Hara and Co., Ltd.). The duration of immobility of the mouse was measured using a video-analysis system (O’Hara & Co., Ltd.) for 6 min.

### Statistical analyses

#### Body weights, blood biochemistry, and behavioral tests

For the behavioral tests, we analyzed the data obtained from each group using one-way or two-way ANOVA. In case of a significant difference detected by ANOVAs, post-hoc analysis (Fisher’s PLSD) was performed.

#### Methylation analysis

For the methylation analysis, microarray data were analyzed using Partek Genomics Suite software (PGS) 6.6 (Partek, Inc.). Data normalization was performed by the default method, including adjusting the probe sequence, RMA Background Correction, Quantile Normalization, and Log (base 2) transformation [23]. Data quality was assessed using PCA and the application of Partek’s quality control workflow. The baseline normalization was performed using the matched-pair normalization tool in PGS, by subtracting the average of IN signals from each IP signal. To detect the MBD binding (methylated) regions in PR or FA relative to CD, we performed a one-way ANOVA and calculated the fold change using geometric mean. Significant region detection (hypermethylation and hypomethylation) was performed using hidden Markov model (HMM) tool in PGS as previously reported [23]. Briefly, in order to capture significant differences in enrichment in PR or FA vs CD across shorter genomic regions (min. ~350 nucleotides) with robust enrichment differences as well as intermediate (min. ~500 nucleotides) and large regions (min. ~1400 nucleotides), three HMM algorithms were applied (s-HMM, m-HMM, and l-HMM). The following cutoffs were used: s-HMM (min. probes: 5, detection states: −5,5, ignore state: 0, max. probability: 0.99, genomic decay: 10000, sigma: 2), m-HMM (min. probes: 10, detection states: −3,3, ignore state: 0, max. probability: 0.99, genomic decay: 10000, sigma: 1), and l-HMM (min. probes: 40, detection states: −1.5,1.5, ignore state: 0, max. probability: 0.99, genomic decay: 10000, sigma: 1).

#### Gene expression

To determine the genes that showed differential expression among the three experimental groups, Tukey’s multiple comparison was performed, and the resultant *P* value was adjusted by Benjamini and Hochberg false discovery rate (BHFDR) to correct multiple tests using the *R* package. Statistical significance was assumed at *P* < 0.0001. The genes that were expressed differently among the groups at the twofold or 0.5-fold change and *P* < 0.0001 level after the BHFDR were regarded as differentially expressed genes. Venn diagrams list of differentially methylated and expressed genes were drawn using the *R* package with the “VennDiagram” function.

## Results

### Body weight and protein-related parameters of recipient mothers

**Fig. 2:**
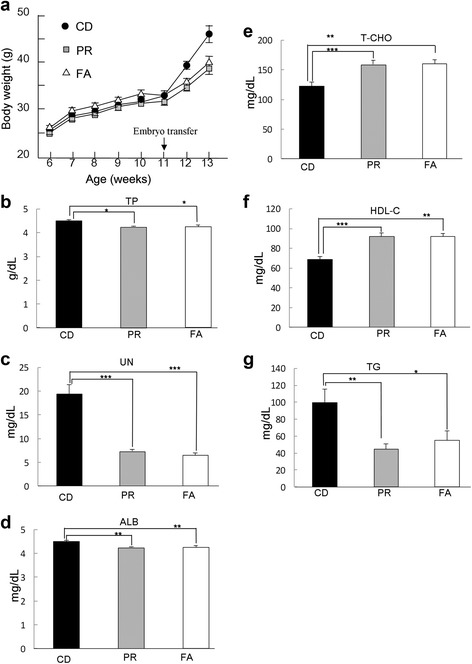
Body weight changes in recipient mothers and serum levels of clinical-biochemical parameters in ICR females. **a** Body weights of recipient mothers is shown. *Arrowhead* indicates the day of embryo transfer. Number of subjects: CD, 11; PR, 14; FA, 14. **b**–**h** Serum levels of clinical biochemical parameters in ICR females. (**b**), total protein (TP); (**c**), urea nitrogen (UN); (**d**), albumin (ALB); (**e**), total cholesterol (T-CHO); (**f**), HDL cholesterol (HDL-C); (**g**), triglyceride (TG) in ICR females that consumed CD, PR, and FA for 1 month are shown. Number of subjects, CD, 7; PR, 7; FA, 6. **a**–**g** *Data* represent the mean ± SEM. *Asterisks* indicate significant differences between groups in post hoc analyses (Fisher’s PLSD test, ***: *P* < 0.0001, **: *P* < 0.01, *: *P* < 0.05)

The experimental design is briefly described in Fig. 1. The three different diets used in this study were protein-restricted (PR), folic acid-supplemented PR (FA), and the control diet (CD) (Table 1). Folic acid is a critical co-enzyme for DNA methylation and may affect epigenetic modification [30]. Oocytes and sperm were obtained from females and males, respectively, which were fed a normal diet (CE-2). In vitro fertilized eggs were transferred to three different kinds of ICR recipient mothers that consumed the PR, FA, or control diet (CD) 1 month before the embryo transfer. Fetuses/neonates were obtained by caesarian section from mothers at 20 days of gestation, and immediately transferred into the care of an ICR foster mother that had consumed the PR, FA, or CD diet. After weaning, offspring consumed the CD. Figure 2a shows average body weights of the recipient mothers of the three different dietary conditions. Similar increases in body weight were observed between the CD and the PR groups at 6–11 weeks of age before ET from B6 mice (two-way ANOVA (Additional file 1)). However, 1 and 2 weeks after the ET (12–13 weeks age), recipient mothers of the PR and FA groups exhibited significantly lower body weights than that of the CD group (two-way ANOVA (Additional file 1)) (Fig. 2a). We performed clinical-biochemical tests in ICR females at 13 weeks of age that consumed experimental diets for 7 weeks. Serum levels of protein-related components such as TP, UN, and ALB (Fig. 2b–d) were significantly lower in the PR and FA groups than in the CD group (one-way ANOVA (Additional file 1)). This may be consistent with differences in body weight. However, as for parameters related to lipid metabolism, levels of total T-CHO and HDL-C were higher in the PR and FA groups than in the CD group (one-way ANOVA (Additional file 1)) (Fig. 2e, f), whereas the TG level was lower (one-way ANOVA (Additional file 1)) (Fig. 2g). We also measured body weights of their progenies from postnatal day 0 and 4 to 12 weeks of age (Additional file 2: Figure S1 A and B). No difference was observed among the three groups with ANOVA, indicating little effect of food components on body weight gain of the progeny (one-way ANOVA (Additional file 1)). In addition, no difference was detected in the performance of embryo transfer among the three groups (Additional file 3: Table S1).

### Effects of maternal-protein restriction on behavioral phenotypes of progeny

**Fig. 3:**
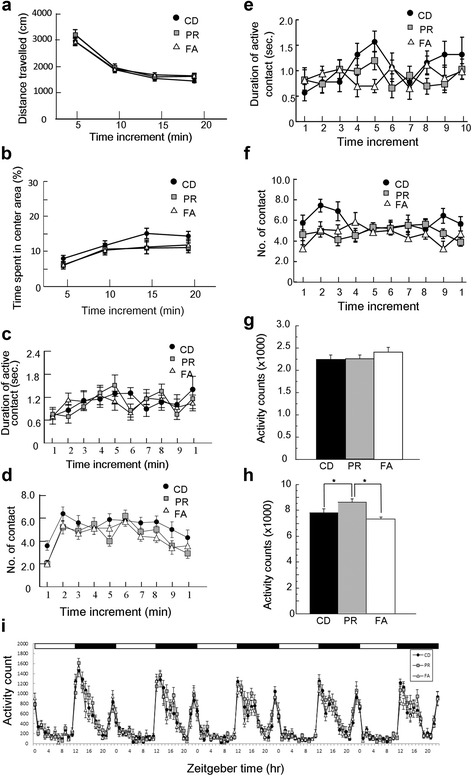
Results of behavioral tests of progenies. **a**, **b** Results of open-field test at 7 weeks of age. **a** Locomotor activity patterns during the open-field test at 7 weeks of age. **b** Time spent in the center (30% of the open-field arena) at 7 weeks of age. **c**, d Results of object-exploration test. **c** Time spent exploring a novel object. **d** Number of times mice that made contact with a novel object. **e**, **f** Results of social-interaction test. **e** Time spent exploring a novel mouse. Number of subjects: CD, 9; PR, 10; FA, 7. **f** Number of times mice made contact with a novel mouse. **g**–**i** Locomotor activity of mice in their home cages. **g** Mean locomotor activity during the light period. **h** Mean locomotor activity in the home cage during the dark period. **i** Locomotor activity in the home cage during the light/dark cycle. **a**–**i** Number of subjects: CD, 10; PR, 10; FA, 7. *Data* represent the mean ± SEM. *Asterisks* indicate significant differences between groups in post hoc analyses (Fisher’s PLSD test, *: *P* < 0.05)

Several tests were performed to examine the behaviors of progenies, and some showed significant changes due to the maternal metabolic changes described above. The open-field test examining locomotor activity in novel place, an indicator for fear/anxiety-like behavior, showed differences between the study groups. We failed to detect changes in the distance that mice traveled in the open-field arena among the three different progenies (two-way ANOVA (Additional file 1)) (Fig. 3a) but found shorter times or durations spent in the center area (30% of the whole arena) in the PR and FA groups than that in the CD group of mice at 7 weeks of age (two-way ANOVA (Additional file 1)) (Fig. 3b). This indicates increased fear/anxiety like behavior in the PR and FA groups [31]. The object-exploration test examining novelty seeking behavior also showed differences between the study groups. In this test, the duration of active contacts to a novel object was decreased in the PR group relative to that of the CD group (two-way ANOVA (Additional file 1)) (Fig. 3c). In addition, the number of contacts with the object was significantly decreased in the PR and FA groups (two-way ANOVA (Additional file 1)) (Fig. 3d). The social interaction test examining prosocial behavior of mice found that the PR and FA groups exhibited shorter durations of active contacts with a novel mouse than that of the CD group (two-way ANOVA (Additional file 1)) (Fig. 3e), as well as decreased number of contacts (two-way ANOVA (Additional file 1)) (Fig. 3f).

We failed to detect differences in the other tests, such as fear-related behavior in a light/dark-transition test (one-way ANOVA (Additional file 1)) (Additional file 4: Figure S2A–C), contextual and cued learning and memory (one-way ANOVA (Additional file 1)) (Additional file 5: Figure S3A, B), and depressive behavior in a tail-suspension test (one-way ANOVA (Additional file 1)) (Additional file 6: Figure S4) between the three groups. Figure 3g–i shows the effect of supplemented folic acid on locomotor activity, which was measured by the home-cage activity test in a familiar setting and daily activity patterns under a light and dark cycle. The mean activity over 5 days in the home cage is expressed as the activity during the light phase (Fig. 3g), activity during the dark phase (Fig. 3h), and activity patterns during the light/dark cycle (Fig. 3i). During the light phase, no difference was observed in the locomotor activity among all groups (one-way ANOVA (Additional file 1)) (Fig. 3i). In dark phase, the PR group showed higher locomotor activity than that of the CD groups (one-way ANOVA (Additional file 1)) (Fig. 3h), and interestingly, this increase was abrogated by folic acid supplementation (one-way ANOVA (Additional file 1)) (Fig. 3h). No difference, however, was observed in the locomotor activity patterns between PR and FA groups (Fig. 3i).

Collectively, these results suggest that PR diet in recipient mothers, irrespective of FA supplementation, enhances susceptibility to fear/anxiety-like, novelty seeking, and prosocial behaviors of progenies.

### Effect of protein restriction on epigenetic alterations

One may expect to find differences in genomic methylation and gene expression among the three different groups. Thus, we performed genome-wide DNA methylation analysis of the three study groups by MBD-chip. The raw data was analyzed using Partek Genome Suite (PGS) software to detect the significantly differentially enrichment regions, as previously reported (see methods). A total of 292 HMM regions exhibited lower promoter methylation levels in the PR group than in the CD group (Additional file 7), whereas 264 HMM regions were higher in the PR group (Additional file 8). A total of 392 genes significantly differed between the PR and the CD group in methylation level. A total of 272 HMM regions exhibited lower promoter methylation levels in the FA group than in the CD group (Additional file 9), whereas 222 HMM regions were higher in the PR group (Additional file 10). A total of 383 genes significantly differed between the FA and the CD group in methylation level. A total of 169 HMM regions exhibited lower promoter methylation levels in the FA group than in the PR group (Additional file 11), whereas 127 HMM regions were higher in the PR group (Additional file 12). A total of 211 genes significantly differed between the FA and the CD group in methylation level. In the expression array, there were 25 genes with lower expression levels (less than 0.5 fold change and *P* < 0.0001 level after the BHFDR) and 55 with higher expression levels (greater than 2.0 fold change and *P* < 0.0001 level after the BHFDR) (Additional files 13 and 14) in the PR group relative to the CD group. There were 48 genes with lower expression levels (less than 0.5-fold change and *P* < 0.0001 level after the BHFDR) and 81 with higher expression levels (greater than 2.0-fold change and *P* < 0.0001 level after the BHFDR) (Additional files 15 and 16) in the PR group relative to the CD group. There were 2 genes with lower expression levels (less than 0.5-fold change and *P* < 0.0001 level after the BHFDR) and 3 with higher expression levels (greater than 2.0-fold change and *P* < 0.0001 level after the BHFDR) (Additional files 17 and 18) in the FA group relative to the PR group. In addition, differentially expressed genes in the PR and FA groups relative to the CD group did not intersect with differentially methylated genes in the PR and FA groups relative to the CD group.

## Discussion

In the present study, we examined the effect of maternal hyponutrition because of a PR diet on developmental and mental disorders of the progenies by comprehensive phenotyping of behaviors. The examination included seven different assays for fear/anxiety-like, novelty seeking, and prosocial behaviors, as well as others that may be related to AD/HD- and ASD-like behaviors. We found significant differences in the assays for fear/anxiety-like, novelty seeking, and prosocial behaviors, which suggests that environmental factors in the embryo/fetus play an important role in the susceptibility of the progenies to these behaviors. This may be parallel to epidemiological studies showing that environmental or nutritional factors affect behavioral symptoms of AD/HD [32] and ASD [33].

A previous study on diet-induced effects reported an increase in the locomotor activity and a lower birth weight of progeny without weight loss in mothers [9]. However, it was conducted under conditions of natural fecundation that did not exclude confounding variables such as the formation of the egg before insemination, milk composition during lactation, and maternal nursing behavior. To exclude those confounding or additional factors pre and postgestation, we used IVF, which allowed us to examine only the effect of diet-induced environmental changes in utero during implantation and gestation. A similar attempt was performed in a recent study examining a transgenerational effect of diet on metabolic diseases [34]. In addition, we used a PR diet supplemented with folic acid to exclude the effect of folic acid on DNA methylation-mediated epigenetic modification. In fact, Lillycrop et al. reported that FA supplementation prevents epigenetic modification of hepatic gene expression in offspring [8]. As expected, the PR and FA diets caused differences in gene expression and genomic methylation in the brains of adult progenies. However, only a moderate proportion of the differences between PR and CD diets were abrogated by FA supplementation. In addition, the FA group showed a different methylation pattern relative to that of the PR group, whereas adult progenies of FA group exhibited behavioral alterations similar to those in the PR group. These results suggest a moderate contribution of FA to the effects of PR on behaviors. Analysis of recipient mothers showed decreases in protein-related components in serum such as TP, UN, and ALB, as well as the triglyceride levels, which were consistent with a decrease in body weight of the mothers, and increases in levels of total cholesterol, HDL-C, and LDL-C. These changes may underlie environmental changes in utero during implantation and gestation, which could cause susceptibility of the offspring.

Several reports have examined AD/HD and ASD mouse models lacking certain gene functions [35–38]. AD/HD models lacking genes related to the dopaminergic system clearly exhibited a deficiency in sustained attention and elevated locomotor activity [35, 38], whereas ASD models lacking genes related with functions of GABAergic and oxytocin related neurons showed decreased social approach [39] and increased fear/anxiety-like behaviors [40]. These behavioral alterations were observed in this study, but the level of differences was moderate. In a previous report, the progenies of B6 mothers that consumed a protein-restricted diet were found to have a lower birth weight relative to the control group; however, the body weight of the mothers were not altered [9]. In the study, the B6 strain was used for mothers and DBA/2 strain was used for fathers of progenies [9], whereas in the present study we used ICR females as recipient mothers and B6 as the source of the embryo. We provided experimental diets to pre-pregnant and pregnant ICR females, and the females that consumed the PR and FA diets exhibited abnormal metabolism of protein relative to the CD group in a clinical-biochemical test. This did not result in lower body weight of these pre-pregnant females; however, pregnant females of the PR group exhibited significantly lower body weight relative to the CD group. Additionally, the progenies of the PR group did not exhibit lower birth weights. Although the amount of protein in the PR group was sufficient for development in newborn mice, it might have been insufficient for body weight gain in the pregnant females. According to the observed phenotype of mice in our previous study [41], the body mass of the ICR strain is much larger than that of the B6 strain. Thus, accumulated protein source in the ICR females may have compensated for the insufficient protein diet in the fetuses of the PR group. In addition, neonates from the ICR females that were fed experimental diets were nursed by the foster mothers that were fed the CD. IVF and ET, which are assisted reproductive technologies, are thought to induce epigenetic changes in the embryo and to alter the adult phenotype in humans [42]. Hence, these differences in experimental conditions resulted in different behavioral phenotypes exhibited by the progenies in the present and previous studies. Therefore, pre-insemination and postnatal/lactation effects of maternal hyponutrition may play a similarly important part in determining offspring behavioral phenotypes.

As in a previous study [9], the present study used only male mice for the behavioral tests in order to eliminate phenotypic variation in female mice caused by the desynchronization of the estrous cycle. The B6 mouse exhibits sex dimorphisms in several behavioral phenotypes, including motor function in the rotarod test, learning and memory in the Morris water-maze test and the fear conditioning test, and locomotor activity in the open-field test [43]. The moderate effects of maternal protein restriction on behavioral phenotypes of the progenies observed in the present study may be gender-specific phenomena.

The methylation levels of promoters in these genes were not correlated with expression levels of the genes. The epigenetic alterations in the brain of the progenies induced by the maternal-protein restriction were clearly observed in the present study. A causal link between epigenetic and behavioral alterations remains to be investigated.

The results of the present study suggest that although maternal protein restriction during pregnancy is a moderate risk factor for developmental or mental disorders of the progeny, it may be substantiated by interaction with genetic factors, including specific mutations or genetic variations. In addition, pre-insemination and postnatal/lactation effects of maternal hyponutrition may significantly contribute to the phenotype.

To verify this hypothesis, it may be necessary to examine the effect of hyponutritional exposure in utero on known developmental disorder models, such as AD/HD and ASD models, or other mouse strains, and to clarify the additive consequences or interaction for behavioral phenotypes.

## Conclusions

The PR group exhibited fear/anxiety related behaviors and inattention to a novel object or a novel mouse more than those of the control diet group. As might be expected, adult brains of the PR group exhibited differences in patterns of the expression array analysis and of genomic-methylation.

Environmental factors in the embryo/fetus play an important role in susceptibility to alteration of progenies’ behaviors. The present study is, to our knowledge, the first to evaluate the effect of maternal hyponutrition on behavioral phenotypes using reproductive technology.

## Additional files

Additional file 1: Values of the statistical analysis. (PDF 155 kb)
Additional file 2:
**Figure S1.** Body-weight changes in progeny. (A) Body weight of progeny immediately after delivery with caesarean section is shown. Number of subjects: CD, 9; PR, 6; FA, 5. Female neonates were used. (B) Body weight of progeny at 4 to 12 weeks of age is shown. Number of subjects: CD, 10; PR, 10; FA, 7. Male mice were used. Data represent the mean ± SEM. (TIF 147 kb)
Additional file 3:
**Table S1.** Performance of embryo transfer in recipient mothers. Mean litter sizes of offspring from each recipient mother are shown. Twenty-four embryos (2-cell) were transferred to each recipient mother. Results of ANOVA analysis, main effect of diet, F(2, 36) = 0.874, P > 0.42. (DOCX 21 kb)
Additional file 4:
**Figure S2.** Light/dark transition test. (A) The number of transition between the light and dark chambers. (B) Time spent in light chamber. (C) Total distance traveled. (A-C) Number of subjects: CD, 10; PR, 10; FA, 7. Data represent the mean ± SEM. (TIF 179 kb)
Additional file 5:
**Figure S3.** Results of fear conditioning test. (A) Freezing responses pre- and post-training with three tone-shock pairs in box A. Pre-train indicates the freezing levels in box A (the shocking chamber) before the onset of training. Context indicates the freezing level after training. (B) Freezing responses with no tone and during tone in box B. (A, B) Number of subjects: CD, 6; PR, 10; FA, 7; Data represent the mean ± SEM. (TIF 125 kb)
Additional file 6:
**Figure S4.** Tail-suspension test. Percent of immobility time in five minutes is shown. Data represent the mean ± SEM. Number of subjects: CD, 6; PR, 10; FA, 7. (TIF 79 kb)
Additional file 7:
**Supplemental information 1.** HMM regions in which methylation levels were lower in PR group relative to CD group. Table also includes identifiers such as gene symbol, Transcript ID, Distance to TSS, percent overlap with region, precise chromosome location of the region, region length, PGS region ID, markers (number of probes), mean enrichment level across the region (fold enrichment), and HMM state (s-HMM: -5, m-HMM: -3, l-HMM: -1.5). (CSV 25 kb)
Additional file 8: HMM regions in which methylation levels were higher in PR group relative to CD group. Table also includes identifiers such as gene symbol, Transcript ID, Distance to TSS, percent overlap with region, precise chromosome location of the region, region length, PGS region ID, markers (number of probes), mean enrichment level across the region (fold enrichment), and HMM state (s-HMM: +5, m-HMM: +3, l-HMM: +1.5). (CSV 23 kb)
Additional file 9: HMM regions in which methylation levels were lower in FA group relative to CD group. Table also includes identifiers such as gene symbol, Transcript ID, Distance to TSS, percent overlap with region, precise chromosome location of the region, region length, PGS region ID, markers (number of probes), mean enrichment level across the region (fold enrichment), and HMM state (s-HMM: -5, m-HMM: -3, l-HMM: -1.5). (CSV 24 kb)
Additional file 10: HMM regions in which methylation levels were higher in FA group relative to CD group. Table also includes identifiers such as gene symbol, Transcript ID, Distance to TSS, percent overlap with region, precise chromosome location of the region, region length, PGS region ID, markers (number of probes), mean enrichment level across the region (fold enrichment), and HMM state (s-HMM: +5, m-HMM: +3, l-HMM: +1.5). (CSV 19 kb)
Additional file 11: HMM regions in which methylation levels were lower in FA group relative to PR group. Table also includes identifiers such as gene symbol, Transcript ID, Distance to TSS, percent overlap with region, precise chromosome location of the region, region length, PGS region ID, markers (number of probes), mean enrichment level across the region (fold enrichment), and HMM state (s-HMM: -5, m-HMM: -3, l-HMM: -1.5). (CSV 15 kb)
Additional file 12: HMM regions in which methylation levels were higher in FA group relative to PR group. Table also includes identifiers such as gene symbol, Transcript ID, Distance to TSS, percent overlap with region, precise chromosome location of the region, region length, PGS region ID, markers (number of probes), mean enrichment level across the region (fold enrichment), and HMM state (s-HMM: +5, m-HMM: +3, l-HMM: +1.5). (CSV 11 kb)
Additional file 13: List of genes that exhibited lower expression level in PR group relative to CD group in adult brain. Table also includes identifiers such as probe name, PrimaryAccession ID, GeneSymbol, Gene ontology (GO), Gene description, chromosome number, P value in Tukey’s multiple comparison test adjusted by FDR, and mean fold change of expression level. (CSV 11 kb)
Additional file 14: List of genes that exhibited higher expression level in PR group relative to CD group in adult brain. Table also includes identifiers such as probe name, PrimaryAccession ID, GeneSymbol, Gene ontology (GO), Gene description, chromosome number, P value in Tukey’s multiple comparison test adjusted by FDR, and mean fold change of expression level. (CSV 18 kb)
Additional file 15: List of the genes which exhibited lower expression levels in FA group relative to CD group. Table also includes identifiers such as probe name, PrimaryAccession ID, GeneSymbol, Gene ontology (GO), Gene description, chromosome number, P value in Tukey’s multiple comparison test adjusted by FDR, and mean fold change of expression level. (CSV 21 kb)
Additional file 16: List of the genes which exhibited higher expression levels in FA group relative to CD group. Table also includes identifiers such as probe name, PrimaryAccession ID, GeneSymbol, Gene ontology (GO), Gene description, chromosome number, P value in Tukey’s multiple comparison test adjusted by FDR, and mean fold change of expression level. (CSV 27 kb)
Additional file 17: List of genes that exhibited lower expression level in FA group relative to PR group in adult brain. Table also includes identifiers such as Probe name, PrimaryAccession ID, GeneSymbol, Gene ontology (GO), Gene description, chromosome number, P value in Tukey’s multiple comparison test adjusted by FDR, and mean fold change of expression level. (CSV 1 kb)
Additional file 18: List of genes that exhibited higher expression level in FA group relative to PR group in adult brain. Table also includes identifiers such as Probe name, PrimaryAccession ID, GeneSymbol, Gene ontology (GO), Gene description, chromosome number, P value in Tukey’s multiple comparison test adjusted by FDR, and mean fold change of expression level. (CSV 1 kb)

## Abbreviations

DOHaD: Developmental origins of health and diseases
ET: Embryo transfer
IVF: In vitro fertilization
PMSG: Pregnant mare serum gonadotropin
