# Supplementary material for: Protein-restricted diet during pregnancy after insemination alters behavioral phenotypes of the progeny
Source: Genes Nutr. 2017 Jan 19;12:1. doi: 10.1186/s12263-016-0550-2 (PMC5248510; doi:10.1186/s12263-016-0550-2)
Supplement: Additional file 1: — Values of the statistical analysis. (PDF 155 kb) [file 12263_2016_550_MOESM1_ESM.pdf]

Supplemental text 1. ANOVA result of body-weight changes in recipient mothers before embryo transfer.

Two-way ANOVA, effect of age, $F(2, 10) = 46.507$, $P < 0.0001$; effect of diet, $F(2, 5) = 8.198$, $P < 0.005$, Fisher's PLSD, CD vs. PR, $P > 0.15$; CD vs. FA, $P < 0.02$; PR vs. FA, $P < 0.0001$.

Supplemental text 2. ANOVA result of body-weight changes in recipient mothers after embryo transfer.

Two-way ANOVA: effect of age, $F(2, 2)=28.395$, $P < 0.0001$; effect of diet, $F(2, 72) = 28.395$, $P<0.001$; Fisher's PLSD, CD vs. PR, $P < 0.0001$; CD vs. FA, $P < 0.0002$; PR vs. FA, $P > 0.29$.

Supplemental text 3. Results of one-way ANOVA in clinical-biochemical test.

TP, $F(2, 17) = 4.831$, $P < 0.03$; Fisher's PLSD, CD vs. PR, $P < 0.02$; CD vs. FA, $P < 0.02$; PR vs. FA, $P > 0.99$: UN, one way-ANOVA, UN, $F(2, 17) = 34.439$, $P < 0.0001$, Fisher's PLSD, CD vs. PR, $P < 0.0001$; CD vs. FA, $P < 0.0001$; PR vs. FA, $P > 0.6$:ALB, $F(2, 17) = 8.61$, $P < 0.003$, Fisher's PLSD, CD vs. PR, $P < 0.003$; CD vs. FA, $P < 0.003$; PR vs. FA, $P > 0.97$; T-CHO, one-way ANOVA, $F(2, 17) = 9.18$, $P < 0.003$; Fisher's PLSD, CD vs. PR, $P < 0.002$; CD vs. FA, $P < 0.002$; PR vs. FA, $P > 0.89$: HDL, one-way ANOVA, $F(2, 17) = 16.822$, $P < 0.0001$; Fisher's PLSD, CD vs. PR, $P < 0.0001$; CD vs. FA, $P < 0.0002$; PR vs. FA, $P > 0.95$; TG, $F (2, 17) = 6.179$, $P < 0.01$; Fisher's PLSD, CD vs. PR, $P < 0.0004$; CD vs. FA, $P < 0.02$; PR vs. FA, $P > 0.89$.

Supplemental text 4. Results of one-way or two-way ANOVA in body weights of progenies at postnatal day 0 and 4 to 12 weeks of age.

One-way ANOVA on postnatal day 0, $F(2, 17) = 0.759$, $P > 0.48$; two-way ANOVA at 4 to 12 weeks of age, effect of age, $F(2, 16)= 94.391$, $P < 0.0001$, effect of diet, $F(2, 8) = 8.341$, $P > 0.25$.

Supplemental text 5. Results of two-way ANOVA in open-field test.

Locomotor activity patterns, effect of time course, $F(3, 96) = 85.272$, $P < 0.0001$, effect of diet, $F(2, 96) = 1.433$, $P > 0.24$; time spent in the center, effect of time course, $F(6, 96) = 10.386$, $P < 0.0001$, effect of diet, $F(2, 96) = 4.335$, $P < 0.016$, Fisher's PLSD, CD vs. PR, $P < 0.008$, CD vs. FA, $P < 0.03$, PR vs. FA, $P > 0.8$.

Supplemental text 6. Results of two-way ANOVA in object-exploration test.

Time spent exploring a novel object, effect of time course, $F(9, 240) = 1.14$, $P > 0.3$; effect of diet, $F(2, 240) = 7.055$, $P < 0.002$; Fisher's PLSD, CD vs. PR, $P < 0.0004$; CD vs. FA, $P <$

0.014; PR vs. FA, $P > 0.4$.

Number of times mice that made contact with a novel object, two-way ANOVA, effect of time course, $F(9, 240) = 3.251$, $P < 0.02$; effect of diet, $F(2, 240) = 5.149$, $P < 0.007$; Fisher's PLSD, CD vs. PR, $P < 0.006$; CD vs. FA, $P < 0.009$; PR vs. FA, $P > 0.9$.

Supplemental text 7. Results of two-way ANOVA in social interaction test.

Time spent exploring a novel mouse, effect of time course, $F(9, 230) = 0.873$, $P > 0.56$; effect of diet, $F(2, 230) = 4.213$, $P < 0.017$; Fisher's PLSD, CD vs. PR, $P < 0.006$, CD vs. FA, $P < 0.05$; PR vs. FA, $P > 0.5$; number of subjects, effect of time course, $F(9, 230) = 0.764$, $P > 0.64$; effect of diet $F(2,230) = 8.325$, $P < 0.0004$; Fisher's PLSD, CD vs. PR, $P < 0.002$; CD vs. FA, $P < 0.0004$; PR vs. FA, $P > 0.49$.

Supplemental text 8. Results of one-way ANOVA in light/dark-transition test.

Number of transition between the light and dark chambers, effect of diet, $F(2, 24) = 0.993$, $P > 0.38$; time spent in light chamber, $F(2, 24) = 1.643$, $P > 0.21$; total distance traveled, $F(2, 24) = 2.661$, $P > 0.09$.

Supplemental text 9. Results of one-way ANOVA in fear conditioning test.

Pre-train, $F(2, 24) = 1.481$, $P > 0.24$; contextual, $F(2, 24) = 0.117$, $P > 0.89$; with no tone in box B, $F(2, 20) = 1.560$, $P > 0.23$; with tone in box B, $F(2, 20) = 1.689$, $P > 0.20$.

Supplemental text 10. Result of one-way ANOVA in tail suspension test.

Effect of diet, $F(2, 24) = 0.34$, $P > 0.71$.

Supplemental text 11. Result of one-way ANOVA in home-cage activity test.

Activity during the light phase, $F(2,177) = 1.204$, $P > 0.3$; activity during the dark phase, $F(2,177) = 3.898$, $P < 0.03$.; Fisher's PLSD, CD vs. PR, $P < 0.02$, CD vs. FA, $P > 0.8$, PR vs. FA, $P < 0.03$.
