# Supplementary figures and images for: Protein-restricted diet during pregnancy after insemination alters behavioral phenotypes of the progeny
Source: Genes Nutr. 2017 Jan 19;12:1. doi: 10.1186/s12263-016-0550-2 (PMC5248510; doi:10.1186/s12263-016-0550-2)

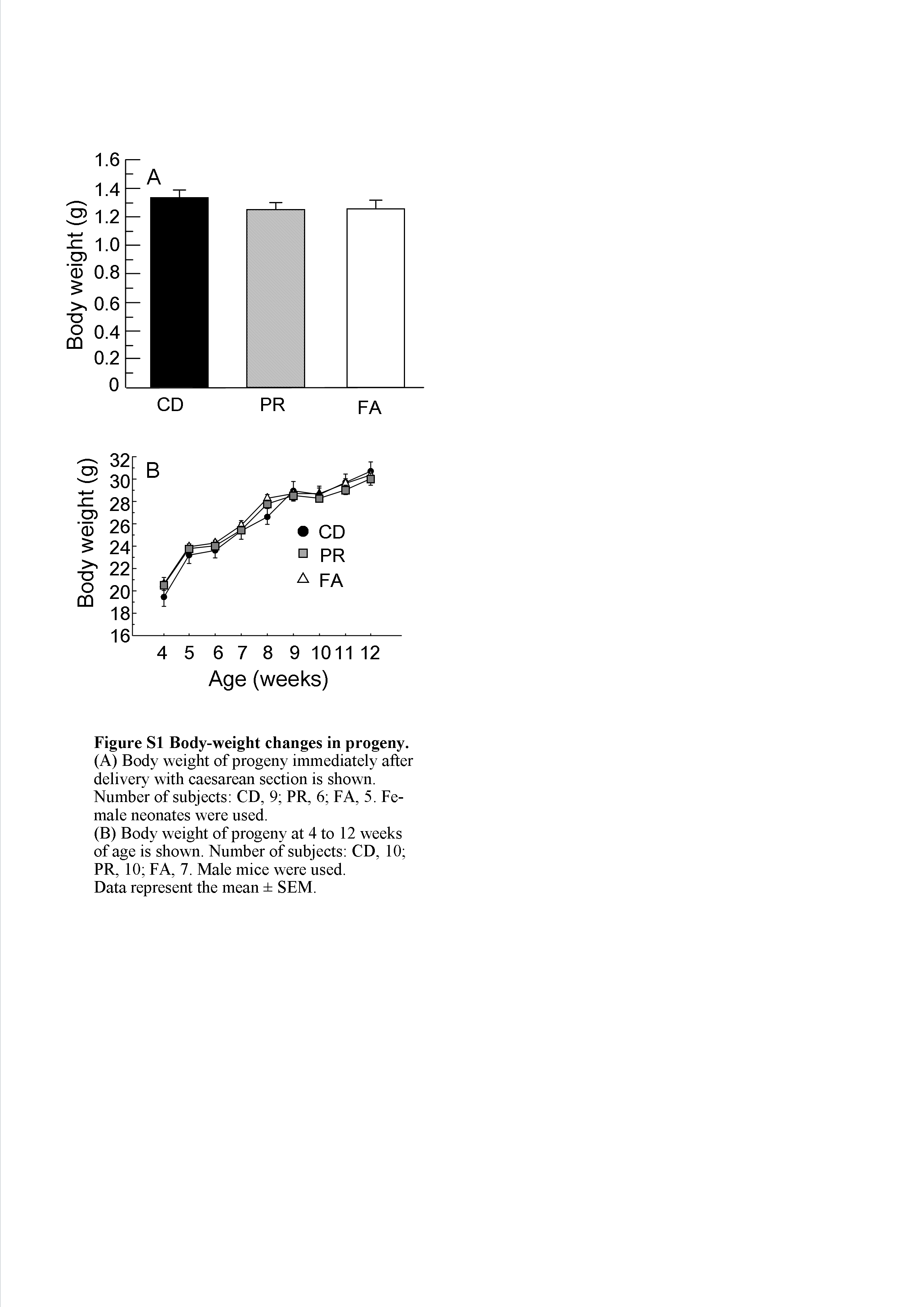

Supplement: Additional file 2: — Figure S1. Body-weight changes in progeny. (A) Body weight of progeny immediately after delivery with caesarean section is shown. Number of subjects: CD, 9; PR, 6; FA, 5. Female neonates were used. (B) Body weight of progeny at 4 to 12 weeks of age is shown. Number of subjects: CD, 10; PR, 10; FA, 7. Male mice were used. Data represent the mean ± SEM. (TIF 147 kb) [file 12263_2016_550_MOESM2_ESM.tif]

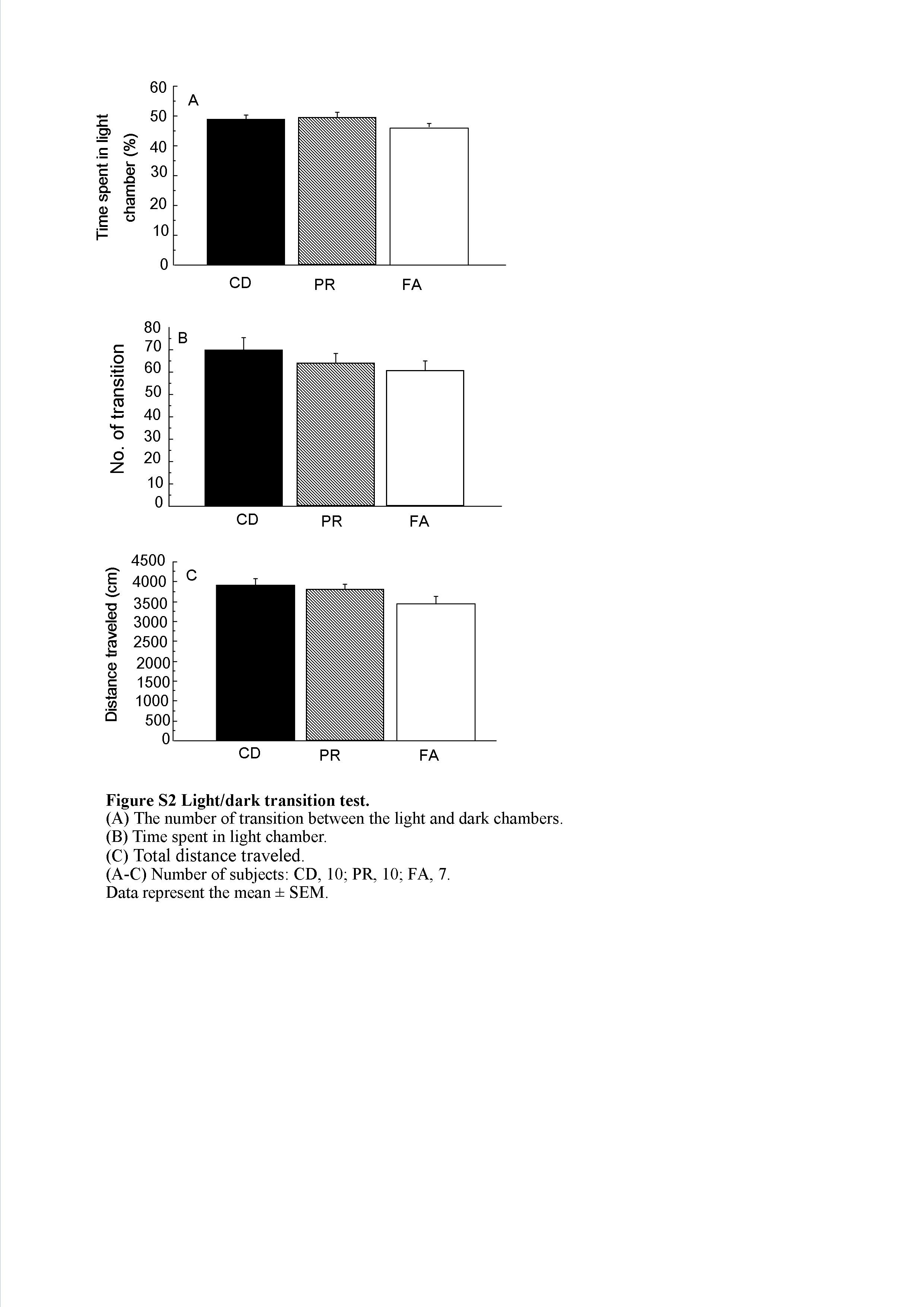

Supplement: Additional file 4: — Figure S2. Light/dark transition test. (A) The number of transition between the light and dark chambers. (B) Time spent in light chamber. (C) Total distance traveled. (A-C) Number of subjects: CD, 10; PR, 10; FA, 7. Data represent the mean ± SEM. (TIF 179 kb) [file 12263_2016_550_MOESM4_ESM.tif]

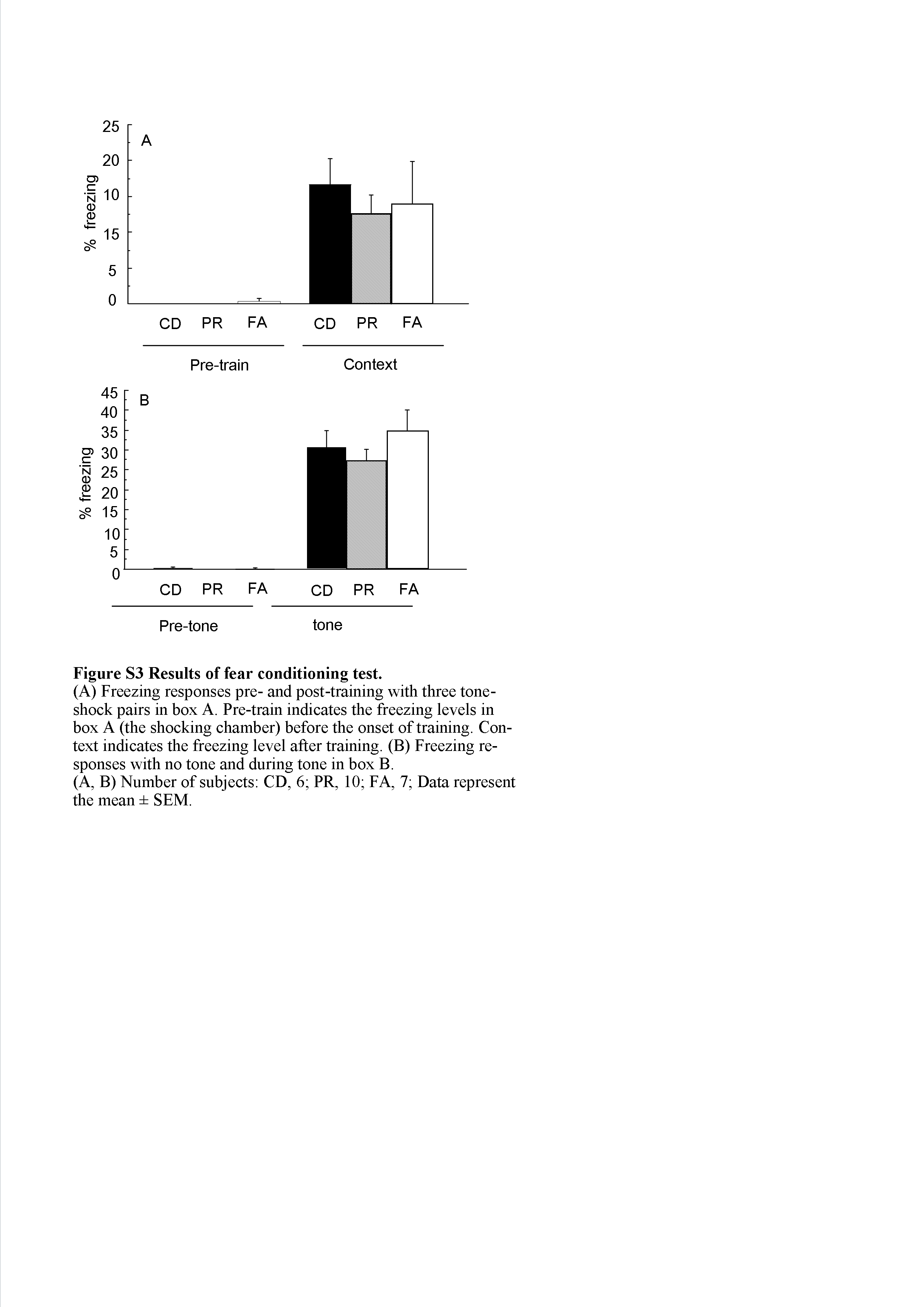

Supplement: Additional file 5: — Figure S3. Results of fear conditioning test. (A) Freezing responses pre- and post-training with three tone-shock pairs in box A. Pre-train indicates the freezing levels in box A (the shocking chamber) before the onset of training. Context indicates the freezing level after training. (B) Freezing responses with no tone and during tone in box B. (A, B) Number of subjects: CD, 6; PR, 10; FA, 7; Data represent the mean ± SEM. (TIF 125 kb) [file 12263_2016_550_MOESM5_ESM.tif]

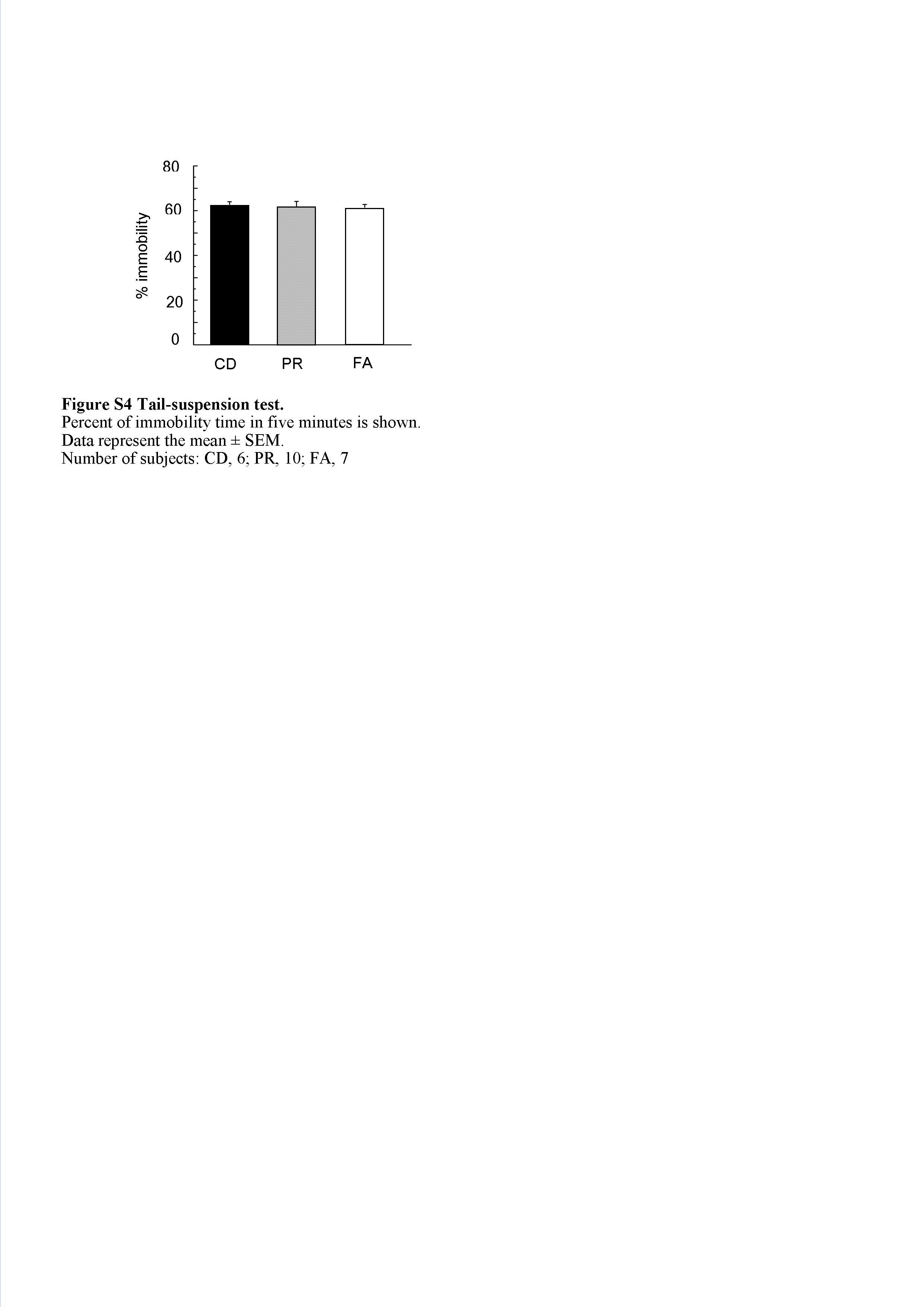

Supplement: Additional file 6: — Figure S4. Tail-suspension test. Percent of immobility time in five minutes is shown. Data represent the mean ± SEM. Number of subjects: CD, 6; PR, 10; FA, 7. (TIF 79 kb) [file 12263_2016_550_MOESM6_ESM.tif]
