# Supplementary material for: Protein-restricted diet during pregnancy after insemination alters behavioral phenotypes of the progeny
Source: Genes Nutr. 2017 Jan 19;12:1. doi: 10.1186/s12263-016-0550-2 (PMC5248510; doi:10.1186/s12263-016-0550-2)
Supplement: Additional file 3: — Table S1. Performance of embryo transfer in recipient mothers. Mean litter sizes of offspring from each recipient mother are shown. Twenty-four embryos (2-cell) were transferred to each recipient mother. Results of ANOVA analysis, main effect of diet, F(2, 36) = 0.874, P > 0.42. (DOCX 21 kb) [file 12263_2016_550_MOESM3_ESM.docx]

Table S1. Performance of embryo transfer in recipient mothers

Diet Litter size (standard deviation) Number of recipients

CD 3.8 (3.4) 11

PR 3.6 (3.1) 14

FA 2.4 (2.3) 14

Mean litter sizes of offspring from each recipient mother are shown. Twenty-four embryos (2-cell) were transferred to each recipient mother. Results of ANOVA analysis, main effect of diet, F(2, 36) = 0.874, *P* > 0.42
